# Infantile Dilated Cardiomyopathy in Alström Syndrome

**DOI:** 10.7759/cureus.74895

**Published:** 2024-12-01

**Authors:** Julien Van Huffel, Emilien Derycke, Thierry Detaille, Stéphane Moniotte, Jelena Hubrechts

**Affiliations:** 1 Congenital and Pediatric Cardiology, Department of Pediatrics, University Hospital Saint-Luc, Brussels, BEL; 2 Pediatric Intensive Care Unit, University Hospital Saint-Luc, Brussels, BEL

**Keywords:** alström syndrome, cardiomyopathy, dilated, genetic testing, infants, rare disease

## Abstract

We report two cases of end-stage dilated cardiomyopathy as the initial manifestation of Alström syndrome (ALMS), in infants aged two and five months. This rare monogenic, autosomal, and recessive genetic condition is a multisystem disorder characterized by visual and hearing impairment, cardiomyopathy childhood obesity, and other anomalies. These cases highlight the importance of genetic testing targeting the ALMS1 gene in the assessment of apparently isolated dilated cardiomyopathy. We review the cardiac features of ALMS. To our knowledge, we also describe the first case of successful surgical pulmonary artery banding in ALMS.

## Introduction

Alström syndrome (ALMS) is a very rare disease (1/1,000,000 live births)[1], characterized by a progressive multisystem impairment. Well-known features are visual disturbance, hearing impairment, cardiomyopathy (CMP), childhood obesity, extreme insulin resistance, accelerated nonalcoholic fatty liver disease, renal dysfunction, respiratory disease, and endocrine and urologic disorders. ALMS is caused by autosomal recessive, bi-allelic, variants in the ALMS1 gene.

In contrast to other types of CMP, dilated CMP (dCMP) is more rarely of genetic origin. We presented two pediatric cases with dCMP as the initial manifestation of ALMS. Moreover, we described the first patient with ALMS who underwent successful surgical pulmonary artery banding for severe heart failure. 

## Case presentation

Case 1

The first case is a two-month-old Caucasian girl who was transferred to our pediatric intensive care unit with cardiogenic shock. She was the firstborn of apparently healthy parents and had an uneventful neonatal period.

**Figure 1:**
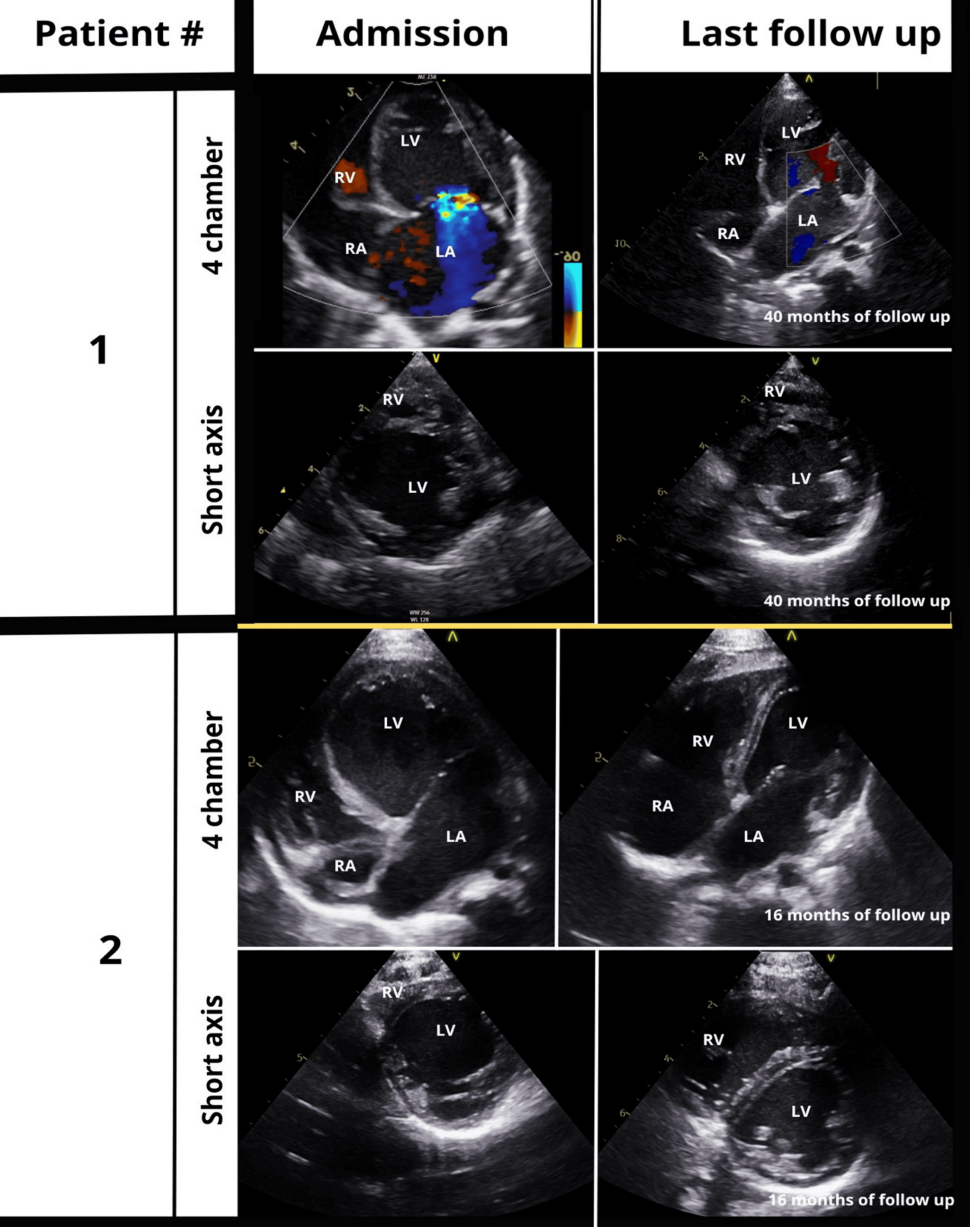
End-diastolic images from four-chamber and short parasternal axis views of patients #1 (Case 1) and #2 (Case 2), acquired at admission and the last follow-up. The gradual improvement of left atrium (LA) and left ventricle (LV) dilation is highlighted. In patient #1, a color Doppler signal was added to show the important mitral valve regurgitation at admission, graded 3/ 4 with a jet area >40% of the left atrium. At 40 months of follow-up, the mitral regurgitation was trivial. The volumes of the right atrium (RA) and right ventricle (RV) were normal in both patients.

Transthoracic echocardiography showed severe left ventricular (LV) dilation with an end-diastolic diameter of 29 mm, corresponding to a +3.8 Z-score according to the Boston Children’s Hospital reference values, LV dysfunction with an ejection fraction of 33%, and severe mitral valve regurgitation (Figure 1).

**Figure 2:**
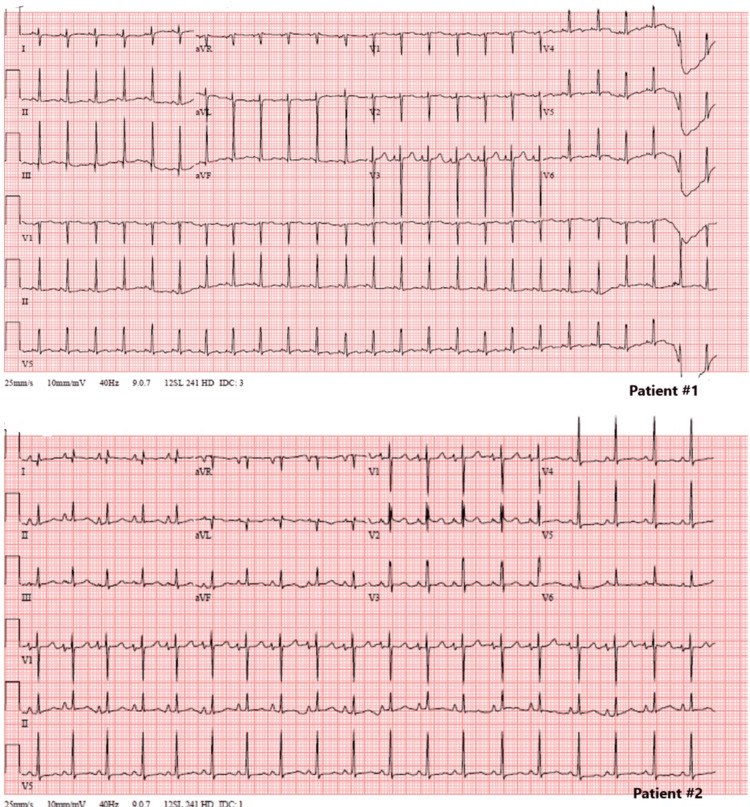
Electrocardiogram of both patients at admission. In patient #1, the ECG showed a normal sinus rhythm at 150/min, with normal atrioventricular conduction (PR interval of 98 ms) and small QRS complexes (58 ms). The QRS axis was normal, at 98°. There were no electrical signs of atrial or ventricular hypertrophy, and no ST depression, strain, or T-wave abnormalities. In patient #2, the ECG showed a normal sinus rhythm at 115/min. Biphasic P-waves in V1 marked left atrial enlargement. Normal atrioventricular conduction (PR interval of 130 ms) and small QRS complexes (64 ms). The QRS axis was normal, at 71°. There was no electrical sign of ventricular hypertrophy, and no ST depression, strain, and T-wave abnormalities.

**Figure 3:**
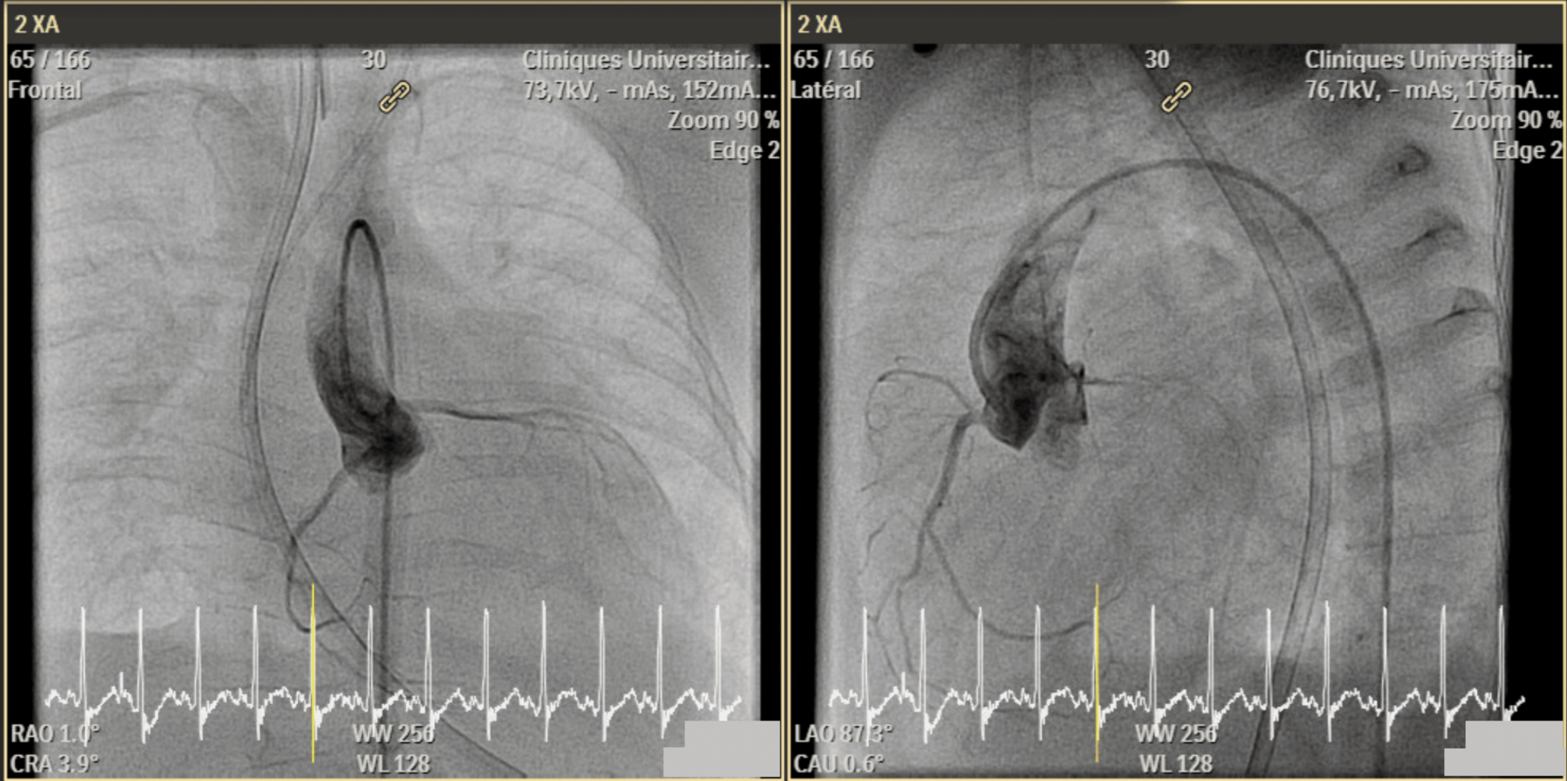
Angiocardiographic images of patient #1, in face and profile. Access was gained from the right femoral artery to the descending and then ascending aorta. Contrast injection through a 4Fr Impress® (Merit Medical Systems, Inc., South Jordan, UT) diagnostic peripheral catheter, placed over a standard 0.035” guidewire, showed normal implantation of two distinct coronary arteries from the left and right aortic sinuses.

Chest X-ray revealed massive cardiomegaly and pulmonary edema. The N-terminal pro-B-type natriuretic peptide (NT-proBNP) level was extremely high, exceeding 70,000 pg/mL (normal value <130 pg/mL). Troponin level was increased up to 303 ng/L (normal value <10 ng/L). The CK-MB level was normal at 21 µg/L (normal value <4.88 µg/L). Electrocardiogram (ECG) was normal for age (Figure 2). Abnormal coronary implantation was ruled out by angiocardiography (Figure 3).

Initial etiological assessment included screening for metabolic diseases, lysosomal/storage disorders, infectious diseases, and selenium deficiency. All results were negative. A first genetic panel for non-syndromic CMP was also performed, with no pathological variants in the tested genes. Cardiologic check-ups of the parents excluded CMP and further family history was unremarkable.

According to current recommendations, management of initial cardiogenic shock included the use of inotropic support (6 days of milrinone, 24 h of dobutamine, and 5 levosimendan cures) and mechanical ventilation (6 days of invasive ventilation followed by 2 days of noninvasive ventilation). Anti-congestive heart failure (HF) treatment by diuretics (loop diuretics, thiazide, and aldosterone antagonists) and vasodilators (angiotensin-converting enzyme [ACE] inhibitors) was optimized. In the absence of diagnosis in the first stage, acute myocarditis could not be excluded and a trial therapy by immunoglobulins was performed. While waiting for the metabolic results, a vitamin complex and carnitine were added to the treatment.

After appropriate HF treatment, LV function recovered to an ejection fraction of 50% and a mildly dilated end-diastolic diameter (+2.4 Z-score).

One month later, at three months of age, she developed abnormal eye movements, including horizontal and vertical nystagmus. Since clinical suspicion was raised by the features of early-onset infantile cardiomyopathy with nystagmus, next-generation sequencing (NGS) targeting the ALMS1 gene (ALMS protein) was performed. The analysis revealed a heterozygous variant in the ALMS1 gene (c.6565_6568del p (Ser2189Metfs*15) maternally inherited and c.1229_1230del p.(Tyr410Phefs*10) paternally inherited. This finding confirmed the diagnosis of ALMS.

At three-year follow-up, the patient now benefits from multidisciplinary care. The cardiac clinical condition is stable under conventional treatment for dCMP, consisting of a beta-blocker (carvedilol 0.3 mg/kg/day), a mineralocorticoid antagonist (spironolactone 2 mg/kg/day), and an ACE inhibitor (enalapril 0.2mg/kg/d). Vision impairment is treated with corrective glasses. So far, there are no other signs of ALMS.

Case 2

**Figure 4:**
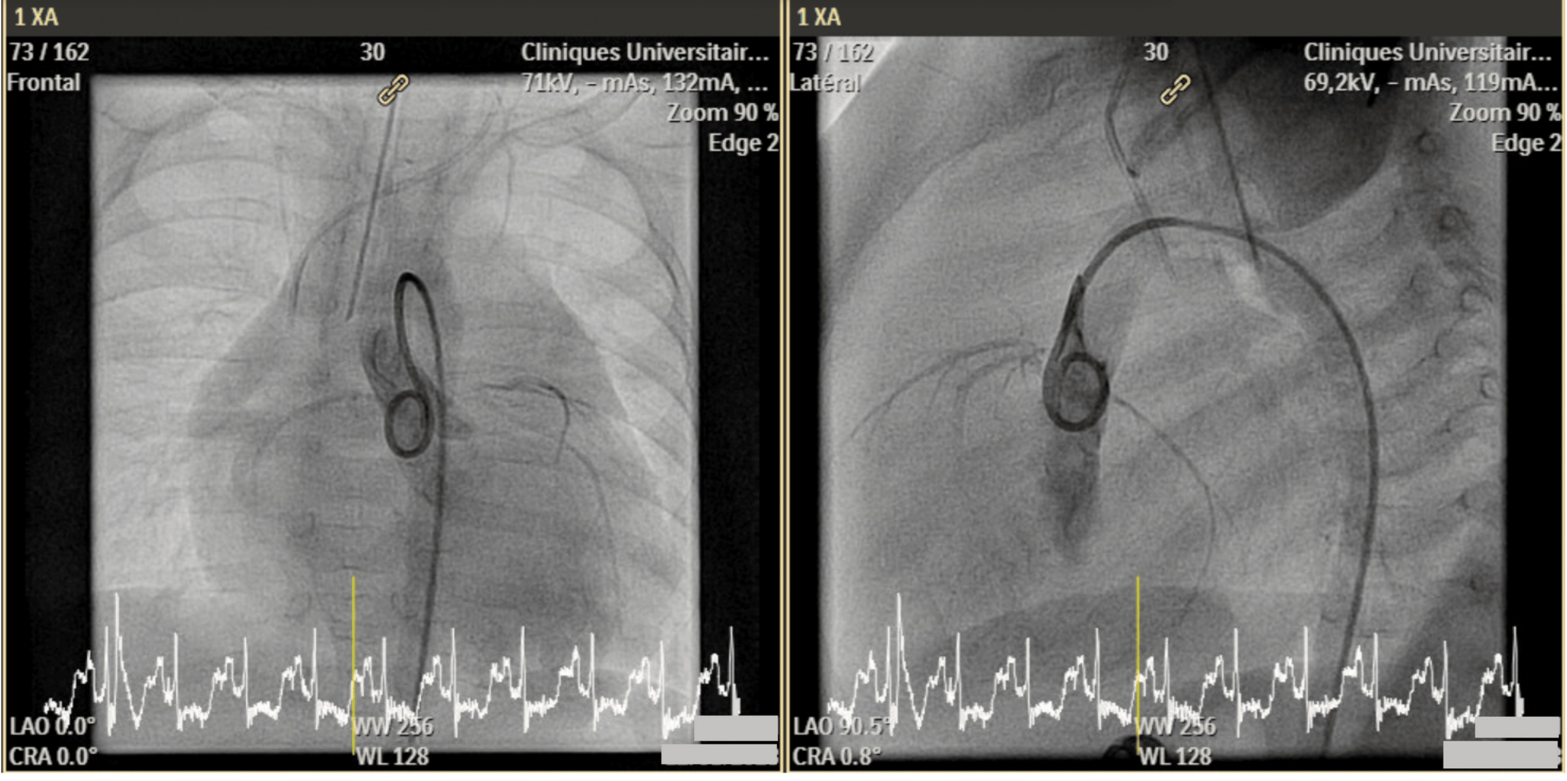
Angiocardiographic images of patient #2, shown in both face and profile views. Access was gained through the right femoral artery to the descending and then ascending aorta. Contrast injection through a 4Fr Performa® (Merit Medical Systems Inc., South Jordan, UT) pigtail catheter showed normal implantation of two distinct coronary arteries from the left and right aortic sinuses. Additionally, the left aortic arch and division of the brachiocephalic branches were normal, with no evidence of coarctation.

The second case is a five-month-old girl, the third child of Asian, non-consanguineous parents, who was referred with acute HF due to dilated cardiomyopathy (dCMP), showing an LV end-diastolic diameter of 45 mm (+10.4 Z-score) on transthoracic echocardiography. Severe LV dysfunction, with an ejection fraction of 28%, and severe mitral and tricuspid valve insufficiency were observed. Chest X-ray showed cardiomegaly with pulmonary edema. ECG showed left atrial enlargement, without ventricular hypertrophy, strain pattern, or pathological T-waves (Figure 2). The NT-proBNP level was also >70,000 pg/mL. CK-MB level was normal at 27.12 µg/L, and the troponin level slightly elevated to 128 ng/L. Neurological examination, including eye movement, was normal. Abnormal coronary implantation was ruled out by angiocardiography (Figure 4).

Importantly, the second offspring of the couple, a female child, had died of sudden death at 18 days of age. Autopsy had demonstrated a nonspecific CMP. At the time, no genetic analyses were performed.

We conducted the same etiological assessment as before. Metabolic screening showed a very high level of urinary carnitine, suggesting a carnitine uptake defect (CUD). A first round of genetic testing was performed, including the same CMP panel and based on the experience of the first case, also targeted testing of the ALMS1 gene. Moreover, SLC22A5 gene targeted testing was added, which ruled out the diagnosis of CUD. Analysis of the ALMS1 gene revealed two pathogenic variants: one class V pathogenic variant, c.5541_5542insGT; p.(Asn1848Valfs*24), maternally inherited, and one likely pathogenic class IV variant, c.9536G>A; p.(Arg3179Gln), paternally inherited. The variant p.(Asn1848Valfs*24) has not been reported previously in patients with ALMS.

**Figure 5:**
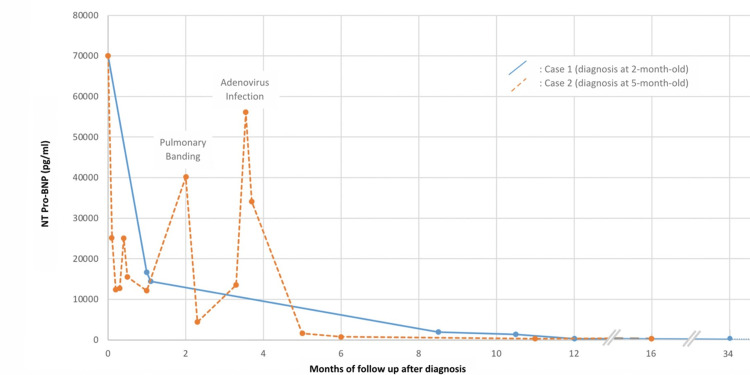
Graphical representation of NT-proBNP levels in both patients as a timeline of their evolution. NT-proBNP, N-terminal pro-b-type natriuretic peptide

This patient needed no days of noninvasive ventilation and inotropic support by milrinone (eight days) and levosimendan (two cures). Despite optimal pharmacologic treatment for congestive HF (ACE inhibitor, beta-blocker, mineralocorticoid antagonist, and thiazide diuretic), the patient was readmitted to the PICU at seven months of age due to overt HF. At that stage, she could not be weaned from respiratory support. We performed reversible pulmonary artery banding to support functional recovery of the dilated LV. This resulted in clinical improvement in our patient, who was then discharged home three weeks later. Two months later, she was briefly re-hospitalized with signs of decompensation, due to adenovirus infection (Figure 5).

Since then, the ejection fraction has gradually recovered to 72%. Currently, general development is normal. She presents normal growth and was weaned off enteral tube feeding during the last seven months. Cardiac treatment was weaned progressively and now consists of a beta-blocker (carvedilol 0.22 mg/kg/day) and an ACEI (enalapril 0.2 mg/kg/day). At 21 months of age, pulmonary artery banding was successfully dilated percutaneously without relapse of HF. At present, dCMP is the only clinical feature of ALMS in this patient.

## Discussion

ALMS is a very rare syndrome (1/1,000,000 live births) with well-known features. Visual impairment is the most consistent manifestation of ALMS and often the first symptom, appearing between a few weeks and six months of age, with an incidence of almost 100%. Hearing loss is the second most common feature, present in 70% of patients before the age of 10.

CMP, responsible for congestive HF, occurs in 42% of infants with ALMS [2]. This incidence is probably underestimated as mortality of severe CMP in infants without etiological diagnosis is significant. The onset, progression, and outcome of ALMS-associated CMP are variable. When it occurs in infants younger than four months old, HF is often severe but transient. Most patients with infantile-onset CMP (before one year) present apparent recovery of cardiac function within two to three years. However, in almost all patients, residual impairment of cardiac function is permanent [3]. The risk of recurrence is described in 1 out of 5 patients. In our two patients, ejection fraction recovered to the low normal range, with no recurrence of HF at, respectively, three years and 22 months of follow-up.

ALMS-associated CMP is typically dilated, nonsegmental with predominantly biventricular involvement [4]. Left ventricular and atrial dilation is observed without reduction of ejection fraction and may be an early stage of CMP. On the other hand, reports show that reduced ejection fraction may be present with normal ventricular size [3]. Cardiac magnetic resonance imaging in ALMS patients shows patchy mid-wall gadolinium delayed enhancement, suggesting an underlying progressive fibrotic process [5]. The mechanism of fibrosis is unknown but seems to play an important role in the development of late CMP. However, for the acute infantile CMP in ALMS, histological findings show signs of mitogenic CMP and suggest a different pathophysiology [3].

Routine cardiac follow-up of patients with ALMS is recommended yearly, or as per clinical need. Transthoracic echocardiography is the gold standard in diagnosing cardiovascular disease in this patient population. Baseline plasma concentrations of natriuretic peptides are useful too [6], among others, to monitor treatment effectiveness or to predict adverse outcomes.

There is no difference in the treatment of congestive HF between ALMS and non-ALMS patients. It includes ACE inhibition and a beta-blocker, titrated to the maximum tolerated evidence-based dose, followed by a mineralocorticoid antagonist [1]. Other molecules such as sacubitril-valsartan or ivabradine should be considered on a case-per-case basis. Regarding the risk of arrhythmia and the need for an implantable cardioverter-defibrillator, standard guidelines as developed by the European Society of Cardiology should be followed [7].

In very young infants, pulmonary artery banding, first described by Schranz et al. [8], is an additional valuable treatment for severe dilated CMP, on the condition that right ventricle function is preserved. The rationale of banding in dCMP is to promote ventricular-ventricular positive interactions and supposed molecular crosstalk, activating the repair potential of the myocardium. By generating an acute leftward shift of the interventricular septum, pulmonary artery banding determines a reshaping of the dilated LV, together with amelioration of LV preload and its intrinsic contractility. In our second case, a seven-month-old girl, we demonstrated the efficiency of this technique. To our knowledge, this is the first publication of pulmonary artery banding in ALMS.

The question of considering ALMS patients with end-stage HF for acute mechanical circulatory support is hard to answer as this has not yet been offered as a destination therapy, in particular in pediatrics. Cardiac transplantation is questionable because of donor organ shortage and multi-organ dysfunction in ALMS. However, successful heart transplantation has recently been published by several authors [9], even in very young infants (<1 year old) [10,11] as a combined heart-lung transplantation [12]. Eliminating heart dysfunction has prevented or even reversed other organ failures.

From a future perspective, it would be interesting to use advanced imaging techniques (e.g., 2D feature tracking and global longitudinal strain) to obtain better documentation of sub-clinical left ventricle dysfunction in this population. With an early diagnosis of persistent subclinical CMP, which could become overt after an insult and manifest as clinical HF, it may be possible to improve cardiac management of certain ALMS patients, for example, by maintaining intensive control of cardiac risk factors.

In our opinion, as it is difficult to differentiate syndromic from non-syndromic cardiomyopathy in newborns and infants, genetic analysis should not be limited to a targeted panel of genes associated with non-syndromic cardiomyopathy, but should also include a broad screening of known genes for syndromic cardiomyopathy, including ALMS1. Early diagnosis improves a patient’s quality of life and longevity by preventing the occurrence of chronic complications as soon as possible. For example, endocrine features in ALMS, such as obesity, type 2 diabetes, dyslipidemia, growth hormone deficiency, or hypothyroidism, may have better outcomes by early treatment. Early diagnosis with adequate genetic counseling leading to a multidisciplinary approach for the patient and his family seems to us the way to go.

## Conclusions

Because ALMS is a very rare and complex syndrome, the diagnosis based on *apparently isolated* dCMP is challenging. However, early diagnosis can result in an early multidisciplinary approach and improve patients’ quality of life. These cases highlight the importance of targeting the ALMS1 gene in the assessment of dCMP. Regarding therapeutic strategies of severe dCMP in very young ALMS patients, we were able to demonstrate that surgical pulmonary artery banding is effective with functional recovery in a seven-month-old girl with HF.
